# The HOPE4MCI study: AGB101 treatment normalizes aberrant network activity and slows entorhinal cortex atrophy in patients with amnestic mild cognitive impairment due to Alzheimer's Disease who are ApoE‐4 non‐carriers

**DOI:** 10.1002/alz70856_100819

**Published:** 2025-12-25

**Authors:** Arnold Bakker, Marilyn S. S. Albert, Sharon Rosenzweig‐Lipson, Richard Mohs

**Affiliations:** ^1^ Johns Hopkins University School of Medicine, Baltimore, MD, USA; ^2^ Department of Psychiatry and Behavioral Science, Johns Hopkins University School of Medicine, Baltimore, MD, USA; ^3^ John's Hopkins University School of Medicine, Baltimore, MD, USA; ^4^ AgeneBio, Inc., Baltimore, MD, USA

## Abstract

**Background:**

Hippocampal hyperactivity is a pathological hallmark of Alzheimer's disease (AD) and a prognostic indicator for AD progression and clinical cognitive worsening in amnestic mild cognitive impairment (aMCI). Extensive data indicate that hippocampal overactivity reflecting an imbalance in inhibitory and excitatory activity is a driver of neurodegeneration and the spread of tau pathology. AGB101 is a proprietary extended‐release formulation of low dose levetiracetam in the dose range previously demonstrated to normalize hippocampal activity and improve cognitive performance in aMCI. The HOPE4MCI clinical trial used a 78‐week protocol of AGB101 treatment to assess progression in amyloid‐positive patients with MCI due to AD.

**Methods:**

A total of 164 participants who were amyloid positive by PET imaging were randomized to placebo or AGB101 treatment using the Clinical Dementia Rating Scale Sum of Boxes (CDR‐SB) score as the primary outcome measure. MRI scans obtained in the study at baseline and after 78 weeks were analyzed providing volume measures of key structures of the medial temporal lobe relevant to AD progression. Blood samples collected at 78 weeks in the study were also analyzed for plasma biomarkers in those participants.

**Result:**

In a pre‐specified subgroup analysis, ApoE‐4 carriers, showed no effect of treatment on any measure. However, in ApoE‐4 non‐carriers, AGB101 treated participants showed a 40% benefit on the CDR‐SB score compared to placebo. These results were not significant due to the planned limited sample size. However, in the ApoE‐4 non‐carriers AGB101 treatment significantly reduced atrophy of the entorhinal cortex. That reduction in atrophy was significantly coupled with the change in CDR‐SB and with NFL and GFAP plasma biomarkers of disease.

**Conclusion:**

Extensive preclinical and clinical studies show that low dose levetiracetam normalizes aberrant network activity and restores inhibitory and excitatory network balance. The HOPE4MCI clinical trial shows that there is meaningful benefit on the CDR‐SB over 18‐month AGB101 treatment and a significant reduction in neurodegeneration in the non‐carriers of ApoE‐4 with MCI due to AD. These findings support the view that reducing hippocampal hyperactivity confers benefit and further testing of AGB101 in patients with MCI due to AD who are non‐carriers of ApoE‐4 is warranted.

